# Microscopic colitis: controversies in clinical symptoms and autoimmune comorbidities

**DOI:** 10.1080/07853890.2021.1962965

**Published:** 2021-08-09

**Authors:** Istvan Fedor, Eva Zold, Zsolt Barta

**Affiliations:** a Faculty of Medicine, Department of Clinical Immunology, Doctoral School of Clinical Immunology and Allergology, Institute of Medicine, University of Debrecen, Debrecen, Hungary; b GI Unit, Faculty of Medicine, Department of Infectology, Doctoral School of Clinical Immunology and Allergology, Institute of Medicine, University of Debrecen, Debrecen, Hungary

**Keywords:** Microscopic colitis, constipation, autoimmune, allergy

## Abstract

**Background:**

Microscopic colitides are chronic immune-inflammatory bowel diseases. The typical presentation is chronic, watery diarrhoea. Inflammation mostly cannot be visualized *via* macroscopic inspection. The diagnosis thus requires histologic sampling. The clinical picture can vary. New investigations can prove valuable in setting up recommendations.

**Patients:**

A total of 103 patients with microscopic colitis (MC) [28 lymphocytic colitis (LC) 27.2%, 75 collagenous colitis (CC) 72.8%] in the Clinical Centre of the University of Debrecen (tertiary care centre) were included, diagnosed between 1993 and 2020. We aimed for a retrospective analysis characterizing Hungarian MC patients. We sought to compare two subgroups of patients (with either LC or CC). Our investigation focussed on dominant alteration of stool habits, autoimmune and allergic comorbidities. Autoimmune diseases were diagnosed in 39% (40) of the patients, allergic diseases in 26.2% (27) of patients and 22.2% of tested patients had alimentary hypersensitivity to certain foods (18 cases out of 81 tested).

**Results:**

Age of diagnosis was younger in LC (44.5 years, SD: 5.3 vs. 51.9 years, SD: 12.8, difference= 7.4 years *p* = .0151). Autoimmune diseases were equally frequent in the two groups (LC: 10 patients 36%, CC: 30 patients, 40%, difference: 4%, *p* = .7124). Food-linked hypersensitivities were more common in CC (LC: 1 patient, CC: 17 patients). Difference in allergic diseases (asthma, rhinitis, urticaria) did not differ between groups (LC: 6 patients, 21%; CC: 21 patients, 28%, difference: 7% *p* = .4739). One-third of the patients did not complain about chronic diarrhoea. These patients had chronic constipation as the main symptom (34 patients, 33%).

**Conclusion:**

Pre-existing autoimmune and allergic diseases were common in patients with MC. Chronic watery diarrhoea is not experienced in many cases. The absence of certain symptoms should not be used to rule out the condition.

## Introduction

Microscopic colitides (MCs) are chronic immune-inflammatory bowel diseases [1–3]. They are divided into two main subtypes: lymphocytic colitis (LC) and collagenous colitis (CC). An incomplete (not classified) variant was also described. Furthermore, mostly from paediatric cases, there were reports of clear cell colitis [4]. The typical presentation of the disease is protracted, watery diarrhoea. Though not widely recognized, aspecific, subtle macroscopic alterations can be present on the mucosa, visible on endoscopy or traditional imaging techniques [5]. These changes are oedematous bowel wall and the presence of mucosal tears. The recognition of characteristic pathological changes requires biopsy with histologic evaluation. No known specific laboratory markers have been discovered so far.

The disease was described in the 1970s. The exact pathophysiology is not yet elucidated [6]. Several factors thought to be associated with an increased risk of microscopic colitis (MC). Most widely known are medications [proton pump inhibitors (PPIs), HMG-CoA reductase inhibitors (statins), non-steroid anti-inflammatory agents (NSAIDs)] [3,7]. Pre-existing autoimmune diseases could also raise the possibility of developing MC. There were reports on smoking and the increased risk of MC [8–10]. Genetic factors were proposed in the disease risk as well [11,12]. There are shared HLA-alleles with certain autoimmune diseases [12]. The inheritable nature of MC is indicated by the increased incidence in families [13]. The underlying cause of the condition is likely to be multifactorial [2]. The disease is managed effectively with budesonide, which is considered to be the drug of choice [14].

There are no universally accepted guidelines for the treatment of this condition. Every new observation is valuable in setting up recommendations for the clinicians managing these disorders. We would like to share the general characteristics, main clinical symptoms and accompanying autoimmune and allergic diseases encountered in Hungarian patients suffering from MC.

## Materials and methods

We sought to compare the differences between patients (total 103 patients, 67 females, 36 males) with the two different forms of MC (28 patients with LC and 75 with CC). Patients were diagnosed and treated at the University of Debrecen Clinical Centre, a tertiary care centre in Eastern Hungary. All diagnoses were verified *via* biopsy and histologic evaluation by experienced, independent pathologists. Appropriate specialists diagnosed autoimmune and allergic disorders according to accepted professional guidelines.

Reviewing the previous medical documentation of patients with histologically confirmed MC, we determined the age of diagnosis, frequency of accompanying autoimmune and allergic conditions and main complaint of bowel movements. The diagnosis of LC or CC was based on the histologic findings. Two independent pathologists were evaluating the samples. By definition, the diagnosis of LC requires the presence of more than or equal to 20 intraepithelial lymphocytes for every 100 epithelial cells. In CC, the subepithelial collagen band thickness exceeds 10 µm, with mucosal inflammatory infiltrate (lymphocytosis). The findings were summarised in an Excel spreadsheet table.

The extracted data were analysed *via* XLStat Excel addon and Medcalc Software. Differences between the groups (age of onset, autoimmune disorders and the clinical symptom’s correlation with AI diseases) were calculated. The comparison of proportions was done through the chi-square test, as recommended for small sample sizes by Campbell [15]. *t*-test was used for comparing the age of diagnosis of the two groups. The observed characteristics were compared to previous studies. On NCBI PubMed site we searched for terms “Microscopic Colitis”, “Collagenous Colitis”, “Lymphocytic Colitis”. The main focus was on more recent literature. IRB Approval was obtained.

## Results

The general overview of patients is listed in Table 1. The mean age of diagnosis was younger in LC (44.5 years, SD: 5.3 vs. 51.9 years, SD: 12.8, difference= 7.4 years *p* = .0151). This differed significantly between LC and CC. The ratio of genders was more balanced in CC (1.6 to 1 ratio, as opposed to 4.6 to 1 female to male). Constipation was significantly more frequent in the CC group (18% in LC; 39% in CC; *p* = .0452). In LC, the majority of patients (23 individuals, 82%) had chronic watery diarrhoea, and five experienced chronic constipation (18%). 45 (60%) of patients with CC had diarrhoea and a higher percentage (29 patients, 39%) of patients had chronic constipation. The difference between constipation in the groups reached a statistically significant level (*p* = .0452).

**Table 1.** General patient characteristics.

	Lymphocytic colitis	Collagenous colitis
Number of patients	28 (27.2%)	75 (72.8%)
Gender	5 males (18%); 23 females (82%) *p* < .0001	31 males (41.3%); 44 (58.7%) females *p* = .0337
Mean age at diagnosis* difference: 7.4 years, *p* = .0151	44.5 years (SD: 5.3)	51.9 years (SD: 12.8)
Comorbid autoimmunity *p* = .7124	10 patients (36%)	30 patients (40%)
Alteration of stooling	Diarrhoea: 23 (82%) constipation: 5 (18%) ratio: 4.6 to 1	Diarrhoea: 45 (60%) constipation: 29 (39%) ratio: 1.4 to 1

Summarizes the general characteristics of our patients. There were significant differences between groups in the mean age of diagnosis of lymphocytic colitis was 44.5 and 51.9 years with collagenous colitis (significant difference between the groups (7.4 years; *p* = .0151) and the ratio of chronic constipation in the two groups: 18% in lymphocytic colitis, and 39% in collagenous colitis; *p* = .0452).

Autoimmune diseases are listed in Table 2. Forty patients had one or more autoimmune comorbidity. The number of these patients was 10 in the LC group (36%) and 30 in the CC group (40%). The patient groups did not differ in this respect (*p* = .7124). The most prevalent autoimmune disease was Hashimoto thyroiditis (14 cases). Sjögren’s syndrome was also frequently present. Sjögren’s syndrome patients all suffered from chronic constipation. There were only four patients with coeliac disease [gluten sensitive enteropathy (GSE)]. The diagnosis of other autoimmune conditions almost always predated the development of MC. Only one patient had MC recognized before the accompanying AI disease (by three months).

**Table 2.** Autoimmune diseases in microscopic colitis.

Lymphocytic colitis	Collagenous colitis
10 (36%)	30 (40%)
Total: 39% of patients; intergroup difference: 4%, *p* = .7124	
Total autoimmune diseases and percentages	
Hashimoto thyroiditis	14 (35%)
Rheumatoid arthritis (RA)	7 (17.5%)
Sjögren syndrome	7 (17.5%)
Nondifferentiated collagenosis (NDC)	5 (12.5%)
Gluten sensitive enteropathy (coeliac disease) (GSE)	4 (10%)
Systemic lupus erythematosus (SLE)	4 (10%)
Mixed connective tissue disease (MCTD)	1 (2.5%)
Ankylosing spondylitis (AS)	1 (2.5%)
Graves-Basedow thyroiditis	1 (2.5%)
Autoimmune hepatitis (AIH)	1 (2.5%)

Forty patients had other diseases of autoimmune origin. Ten patients in the lymphocytic colitis group (36%) and 30 patients in the collagenous colitis group (40%). There was no significant difference between the groups *p* = .7124).

The co-occurring diseases of allergic-atopic origin (without GI allergies) are found in Table 3. Twenty-seven patients were diagnosed with allergic diseases (26%). 6 (21%) in the LC group and 21 (28%) of CC cases. The difference between the groups was not significant (7% difference, *p* = .4739). Diseases with allergic aetiology were mostly affecting the respiratory tract (bronchial asthma and allergic rhinitis).

**Table 3.** Allergic diseases in microscopic colitis.

	Lymphocytic colitis	Collagenous colitis
Total Difference: 7.4%, *p* = .4739	6 (21.4%)	21 (28%)
Asthma	3 (50%)	9 (42.9%)
Rhinitis	1 (16.7%)	10 (47.6%)
Urticaria	2 (33.3%)	3 (14.3%)
Eczema	0	1 (4.8%)

Twenty-seven patients were diagnosed with allergic diseases (26%). 6 (21%) of the lymphocytic colitis patients and 21 (28%) of collagenous colitis patients. The difference between groups did not reach a statistically significant level (7.4% difference, *p* = .4739). The most frequent diseases with allergic aetiology were affecting the respiratory tract (asthma and allergic rhinitis).

Summary of the presence of food-specific IgE antibodies can be found in Table 4. Eighty-one patients (79%) were tested for alimentary allergies. In most cases (78% of the patients), the test results were negative. 18 patients (22%) had elevated concentrations of IgE class antibodies against certain food antigens. The difference between the two groups was marked: only one person in the LC group was affected by elevated IgE class antibodies against specific food antigens, whereas the other 17 cases were all reported in the CC group.

**Table 4.** Alimentary hypersensitivities.

Foodborne allergy tested	81	78.6%
Negative	63	77.8%
Positive	18	22.2%
Positive IgE against food antigens		
Peanut	8	44.4%
Soy	6	33.3%
Tomatoes	6	33.3%
Milk	3	16.7%
Egg	3	16.7%
Gliadin	1	5.6%
Bananas	1	5.6%
Peach	1	5.6%
Oats	1	5.6%

Summarises the presence of food-specific IgE antibodies. Eighty-one patients were tested for alimentary allergies. 18 patients (22%) had antibodies of the IgE class against certain food antigens. Only one person in the lymphocytic colitis group had a food allergy. The remaining 17 cases were within the collagenous colitis group.

## Discussion

Though our study has its limitations, due to it being a retrospective analysis of available data on two different group of patients, without the inclusion of a healthy control subgroup. Moreover, sample size was also small, still there are some noteworthy findings.

Albeit previous studies reported LC to be more frequent, we were unable to confirm this. Out of our 103 patients, 28 and 75 had the histologic picture of LC and CC, respectively (27.2% and 72.8%; *p* < .0001). The gender ratio revealed female predominance: 67 women and 36 men were diagnosed with either LC or CC (female: 65% vs. male: 35%, *p* < .0001).

In LC female patients outnumbered males by 4.6 times. Women and men had similar representation in the CC group (female to male ratio 1.4 to 1). The findings (the frequency of LC and CC) differ from what was expected [16]. Probably the discrepancy is due to small sample size. There might be different clinical characteristics depending on region and ethnicity [16].

The age at diagnosis was younger than expected among our patients. As LC patients were younger at diagnosis, one might ask: whether the lymphocytic infiltrate can be a forerunner of collagen band thickening. The two conditions might represent the same disease, though in different stages [17]. This concept is not a novelty and was already hypothesized. There is also an overlap in histologic findings in these conditions [18]. MC is rare in young adults (<30 years) and children. Nonetheless, there are few reports on paediatric cases with MC [19–21].

As expected, the proportion of women with autoimmune disorders was higher than in men. 47.8% of women had AI disease and 22.2% of men. AI diseases predated the development of MC. Most of the previous investigations reported this pattern. Autoimmune diseases mostly develop in the young adult-middle aged population. On the other hand, MC is associated with more advanced age.

Coeliac disease (GSE) is reported to be associated with LC, but we were unable to confirm this [22,23]. GSE was only found in 4 out of 103 patients, 3.8%. There was no evidence of either Crohn’s disease or ulcerative colitis on examined biopsy specimens. Thus according to our findings, the presence of MC does not increase the risk for Crohn’s disease or UC.

According to previous literature, the colitis and GI symptoms can develop after the eradication of Helicobacter pylori colonization. Similar reports are available on cases of other immune-mediated gastrointestinal diseases (Crohn’s, UC, GSE) [24–26]. *Helicobacter pylori* infection was described to be associated with extraintestinal autoimmune diseases, though this is not a widely accepted concept [27,28]. Our patients were not tested for *H. pylori* colonisation, thereby we do not have data on its frequency.

Seventy eight of the patients were screened for food-antigen specific IgE antibodies. The increased epithelial permeability in MC possibly favours the development of these antibodies. Most common foodborne allergens were peanuts, soy and tomatoes

Allergic and atopic diseases were also assessed. The intestinal tract and airways share their embryologic origin, and they have a similar basic structure. There are other similarities between asthma and microscopic colitides in general (e.g. lymphocytosis, later collagenous band thickening) [29,30]. None of the patients had pulmonary fibrosis, though there are common variables in the features of PF and CC. This finding is similar to that others have described. None of the patients had systemic sclerosis or CREST syndrome. The development of CC in systemic sclerosis is rare [31–33].

The classic presentation of MC is watery diarrhoea (more than three bowel movements per day, at least 250 g stool of liquid consistency daily, for more than 1 month), but others reported cases characterized by chronic constipation [34]. Inflammation can reduce intestinal peristalsis [3,33]. It is possible, that patients with chronic constipation or alternating stooling habits could have underlying MC. For certain patients the dominating symptom changed during disease course. Periods of diarrhoea were followed by periods of constipation (less than three bowel movements per week, with excessive straining and hard stool, for at least 12 weeks in the last few 12 months).

Thus, symptoms cannot be used to rule out MC. According to our findings, there were no significant differences between those with or without diarrhoea in the frequency of other immune diseases. The proportion of patients with autoimmune comorbidities was 37% in those presenting with diarrhoea, and 44% of those with chronic constipation (*p* = .4972).

We observed that patients with Sjögren’s syndrome all suffered from chronic constipation. Sjögren’s syndrome patients have compromised nutrient processing and absorption, with an inadequate quantity of saliva. Oesophageal dysmotility and gastric hypomotility was also described earlier. Autonomic and enteric nervous system damage is not infrequent in Sjögren’s syndrome. It can contribute to the dysmotility [35,36]. Our observation that some patients had chronic constipation or alternating stooling habits is not surprising [37,38]. Even diseases with dubious pathogenesis (such as irritable bowel syndrome) can present with neuromuscular dysfunctions of the gastrointestinal tract [39].

The role of different inflammatory cytokines in regulating ECM structural homeostasis was described previously [40–42]. There are earlier reports on cases, where prolonged treatment with TNF-α inhibitors—infliximab or adalimumab—, patients developed CC. A possible reason could be TGF-β overactivity due to TNF-α blockade. This might favour excess collagen synthesis and fibrous tissue remodelling. Thus, patients with IBD can develop characteristics of CC after administration of TNF-α inhibitors for longer periods [43]. MC can be present with “classic IBD” in patients, as recently shown by Khalili et al. [44].

The role of transforming growth factor-β (TGF-β) was described in other conditions with excessive fibrous thickening [45–48]. In pulmonary fibrosis and systemic sclerosis, there is evidence for excessive TGF-β activity. No recommendations exist for using anti-fibrotic agents in CC, even though in other fibrotic diseases they can be beneficial and delay disease progression.

## Conclusions

In summary, our findings were not always in agreement with previous reports. We saw a significant number of patients without diarrhoea (one-third of total cases). Autoimmune comorbid diseases preceded the onset of MC. Sjögren’s syndrome was associated with chronic constipation. Though our investigation has the limitation, not including a healthy control group (thereby, the prevalence of other immune-mediated diseases and allergies cannot be compared to that seen in the otherwise healthy individuals), it is worth to compare the two differing groups to each other.

From our observations, symptoms and stooling habits cannot be used to rule out the presence of MC. Not every patient suffers from chronic watery diarrhoea. The risk for autoimmune diseases does not show a marked difference between the two groups. Though microscopic colitides are female-predominant diseases, CC subtype showed a fairly balanced gender distribution. The therapeutic control of the disease was effective with budesonide.

## Ethical approval

All methods were performed following the relevant guidelines and regulations. The study protocol was approved by the local Ethics Committee of Kenezy University Hospital, University of Debrecen, registration number: E/51/2019) and was in full compliance with Good Clinical Practices and the Declaration of Helsinki (1996). By signing a written informed consent, all patients agreed to have the study results regarding any side effects as well as possible risks and benefits of the study published.

## Data Availability

The data that support the findings of this study are available from the corresponding author, [IF], upon reasonable request.
